# Complete genome sequence of *Mucilaginibacter gossypii* P3, a heavy metal(loid)-resistant bacterium, isolated from a gold and copper mine

**DOI:** 10.1128/MRA.00172-23

**Published:** 2023-10-10

**Authors:** Yuan-Ping Li, Xiao-Jun Yang, Yan-Shuang Yu, Guo-Hong Liu, Fajie Feng, Jiang-Hua Ye, Rensing Christopher

**Affiliations:** 1 College of Tea and Food, Wuyi University, Wuyishan, Fujian, China; 2 College of Ecology and Resource Engineering, Wuyi University , Wuyishan, Fujian, China; 3 Institute of Environmental Microbiology, College of Resources and Environment, Fujian Agriculture and Forestry University, Fuzhou, China; 4 Agricultural Bio-resources Research Institute, Fujian Academy of Agricultural Sciences, Fuzhou, Fujian, China; The University of Arizona, Tucson, Arizona

**Keywords:** *Mucilaginibacter*, arsenic, plasticity, heavy metals

## Abstract

*Mucilaginibacter gossypii* P3, which was isolated from the sub-surface soil of the Zijin Gold and Copper Mine, displayed extremely high resistance to multiple heavy metal(loid)s and contained two novel *ars* operons. Complete genome sequencing of P3 yielded a single, closed genome of 7,187,928 bp, with GC content of 42.79%.

## ANNOUNCEMENT

The genus *Mucilaginibacter* was first reported in 2007 by Pankratov et al. (1), with the species type *Mucilaginibacter paludism* isolated from an acidic Sphagnum peat bog (1). Currently, strains belonging to the genus *Mucilaginibacter* have been isolated from a variety of environments, such as fresh water (2), sediment (3), soil (4), marine sand (5), and wood (6). *Mucilaginibacter* is known for producing substantial amounts of extracellular polymeric substances (7), degrading cellulose (8), and acting as a plant growth promoting bacterium under stress (9).

*Mucilaginibacter gossypii* strain P3, isolated by using R2A (DSM medium 830) agar plates containing 2 mM CuSO_4_·5H_2_O from subsurface soil of one of the largest gold mines in Asia (Zijin Gold and Copper Mine) known for a long history of mining, was sequenced and described here (10).

*Mucilaginibacter gossypii* strain P3 stored at −80°C was plated on R2A agar and grown at 28℃. After around 12 h inoculation, a single colony was picked and inoculated into R2A broth and shaken at 28℃, 180 rpm overnight.

Genomic DNA (gDNA) of strain P3 was extracted according to the manufacturer’s instructions using TIANamp Bacteria DNA kit (lot No. Q5727; Tiangen, China). Complete genome sequencing was performed by Biomarker Technology Co., Ltd. (Beijing, China); the Covaris M220 (Covaris, Massachusetts, USA) was used to break the gDNA for building the Oxford Nanopore Technologies library that was constructed by using SQK-LSK109 Ligation Sequencing Kit (lot no. BK-AUXS008, BaiYi, China). Base calling for the raw reads was performed using Guppy v3.2.6 by fast calling (11). Canu v1.5 (12), Pilon v1.22 (13), and Circlator v1.5.5 (14) were used for the assembly, polishing, and circularization, respectively. Default parameters were used for all software unless otherwise specified.

The complete genome sequence of P3 contained 20 genes predicted to encode functions involved in conferring arsenic resistance, located on three *ars* operons and encoding ArsR, MrArsM*_Bact_*, ArsC, ACR3, metallophosphoesterase (MrMe1*_Bact_*), metallophosphoesterase (MrMe2*_Bact_*), Hp, MrArsU*_Bact_,* and ArsN. Within the three *ars* operons, two *ars* operons were identified (*arsR1-mrarsU_Bact_-arsN-arsC1-hp-acr31-mrme1_Bact_-mrme2_Bact_* and *arsR2-mrarsU_Bact_1-arsN1-arsC2-hp2-acr32-mrme1_Bact_1-mrme2_Bact_1*) that displayed a similar composition of genes with the encoded amino acid sequence similarity between the same genes being higher than 69.84%. Moreover, genes such as *mrme1_Bact_* and *mrme2_Bact_* located on the two novel *ars* operons have not been characterized but are widespread in members of the genus *Mucilaginibacter* when looking for homologous proteins on NCBI. Here, we report the complete genome sequence of heavy metal(loid)-resistant *Mucilaginibacter gossypii* strain P3.

The complete genome sequence by Nanopore that was assembled here resulted in a single circular closed genome with a GC content as 42.79%. Sequence annotation for the strain was performed using the NCBI Prokaryotic Genome Annotation Pipeline (15). The annotation predicted 6,209 genes, 6,142 coding sequences, and 67 RNA genes (including 55 tRNAs, 9 rRNAs, and 3 noncoding RNAs). The sequencing data and assembly metrics are shown in Table 1.

**TABLE 1:** Sequencing and assembly data of strain *M. gossypii* P3

Parameter	Strain P3
Genome size (Mb)	7.187928
G+C content (%)	42.79
Total no. of raw reads	139,364
N_50_ value (bp)	16,274
Avg read length (bp)	7862.23

## Data Availability

Genomic raw reads of the strain P3 are deposited in the SRA database under accession numbers SRR25759840 and SRR25759839, and the genome sequence is available at GenBank under accession number GCA_017569325.1, Bioproject PRJNA712965, and Biosample 18237936.
